# Cultivating Lentinula edodes on Substrate Containing Composted Sawdust Affects the Expression of Carbohydrate and Aromatic Amino Acid Metabolism-Related Genes

**DOI:** 10.1128/msystems.00827-21

**Published:** 2022-02-22

**Authors:** Xiying Huang, Runji Zhang, Qinyan Yang, Xinzhu Li, Quanju Xiang, Ke Zhao, Menggen Ma, Xiumei Yu, Qiang Chen, Xianfu Zeng, Lujun Zhang, Petri Penttinen, Yunfu Gu

**Affiliations:** a Department of Microbiology, College of Resources, Sichuan Agricultural University, Chengdu, China; b Chengdu Academy of Agriculture and Forestry Sciences, Chengdu, China; c Shanghai Academy of Agriculture Sciences, Shanghai, China; University of California San Diego

**Keywords:** *Lentinula edodes*, composted sawdust, transcriptomics, proteomics, brown film formation

## Abstract

In mushroom cultivation, composting the substrate can make the nutrients more easily absorbed by hyphae due to the degradation of lignin, cellulose, and other organic matter. However, the effects of cultivating Lentinula edodes on composted substrate and the related molecular mechanisms have not been studied systemically. We applied transcriptomics, qRT-PCR, and proteomics to study L. edodes cultivated on substrates with fresh (CK) and composted (ND) sawdust, focusing on the brown film formation stage. The time of brown film formation was shorter and the mycelium growth rate and crude polysaccharide content of the brown film were higher in ND than in CK. The faster growth rate in ND may have been due to the higher nitrogen content in ND than in CK. Among the 9,455 genes annotated using transcriptomics, 96 were upregulated and 139 downregulated in ND compared with CK. Among the 2,509 proteins identified using proteomics sequencing, 74 were upregulated and 113 downregulated. In the KEGG pathway analyses, both differentially expressed genes and proteins were detected in cyanoamino acid metabolism, inositol phosphate metabolism, pentose and glucuronate interconversions, phosphatidylinositol signaling system, RNA polymerase, starch and sucrose metabolism, and tyrosine metabolism pathways. A large number of differentially expressed genes (DEGs) related to aromatic amino acid metabolic and biosynthetic process were upregulated in ND. Most of the DEGs annotated to carbohydrate active enzymes were downregulated in L. edodes growing on composted sawdust containing substrate, possibly due to the lower hemicellulose and cellulose contents in the composted sawdust. The results suggested that using composted substrate may decrease the cultivation time and improve the quality of L. edodes and revealed the underlying molecular mechanisms.

**IMPORTANCE** Composted substrates are not commonly used in the cultivation of Lentinula edodes, thus the effects of cultivating L. edodes on composted substrate and the related molecular mechanisms have not been studied systemically. We studied L. edodes cultivated on substrates with fresh (CK) and composted (ND) sawdust, focusing on the brown film formation stage, and determined the composting related differences in the substrate and in the growth and gene expression of L. edodes. Cultivation on composted substrate was beneficial and showed potential for decreasing the cultivation time and improving the quality of L. edodes. Analyzing the expression levels of genes and proteins in brown film revealed gene and metabolism pathway level changes that accompanied the cultivation on composted substrate.

## INTRODUCTION

Lentinula edodes (shiitake mushroom) has high nutritional and economic value (1), and its production accounts for more than one fifth of the mushroom production worldwide (2). The growth of L. edodes proceeds from hyphal knot growth to brown film formation by the mycelia, primordium initiation, and fruiting body development (3). Out of these, the brown film formation, i.e., formation of a brownish red, elastic protective layer, is a unique stage in L. edodes growth cycle. Successful cultivation of L. edodes depends on the quality of brown film because the development of primordium and the quality and quantity of fruiting body are affected by the depth and thickness of the brown film (4).

L. edodes is grown on sawdust, hazelnut husk (5), ground wheat straw (6), and bagasse enriched with rice bran and sugarcane molasses (7) substrates. In the large-scale cultivation of L. edodes, sawdust is still the most widely used main substrate. The substrates are generally mixed and sterilized. Appropriate adjustment of culture substrate is helpful to improve the potential nutritional value of edible fungi (8). Composting the substrates, which is often used in cultivating *Agaricus bisporus* (9), can decrease the numbers of pathogenic microorganisms (10) and miscellaneous bacteria pollution. In addition, due to the degradation of lignin, cellulose, and other organic matter (10, 11), nutrients are more easily absorbed by hyphae from the composted substrate (12, 13). However, composted substrates are not commonly used in the cultivation of L. edodes. Composting accelerated the growth rate of L. edodes mycelium (14). To our knowledge, the other effects of the cultivating L. edodes on composted substrate and the related molecular mechanisms have not been studied systemically. Therefore, we studied L. edodes cultivated on fresh and composted sawdust, focusing on the brown film formation stage. Differences in enzyme activity and gene expression at RNA and protein levels were analyzed using traditional enzyme assays, transcriptomics, and proteomics. The expression of differentially expressed genes were validated using qRT-PCR.

## RESULTS

### Composting changed the quality of the sawdust.

Composting changed the color of sawdust from brownish red to dark brown. Cellulose, lignin, hemicellulose, and organic carbon contents were lower, and total nitrogen content was higher in the composted (ND) than in the not-composted (CK) sawdust (*P* < 0.05) (Table 1). The growth rate of L. edodes mycelia was faster and the polysaccharide content of the brown film was higher in ND than in CK (*P* < 0.05) (Table 1). In addition, the brown film formation started earlier and was completed 14 days earlier in ND than in CK, showing that sawdust composting promoted the growth of L. edodes.

**TABLE 1:** Physicochemical characteristics of fresh (CK) and composted (ND) sawdust, and the mycelium growth rate and polysaccharide content of the brown film of L. edodes cultivated on substrate containing CK and ND^*a*^

	CK	ND
Lignin (%)	16.62 ± 0.05 A	12.97 ± 0.44 B
Cellulose (%)	38.16 ± 0.31 A	28.46 ± 0.21 B
Hemicellulose (%)	28.17 ± 0.50 A	17.88 ± 0.38 B
Organic carbon (%)	44.04 ± 0.86 A	35.42 ± 1.19 B
Total nitrogen (%)	0.40 ± 0.01 B	1.36 ± 0.12 A
Growth rate (cm d^−1^)	0.23 ± 0.04 B	0.32 ± 0.02 A
Polysaccharide (%)	6.47 ± 0.38 B	9.26 ± 0.28 A

a Different superscript letters in a row indicate statistically significant difference (*P* < 0.05, *n* = 3).

### Transcriptome analysis.

**FIG 1:**
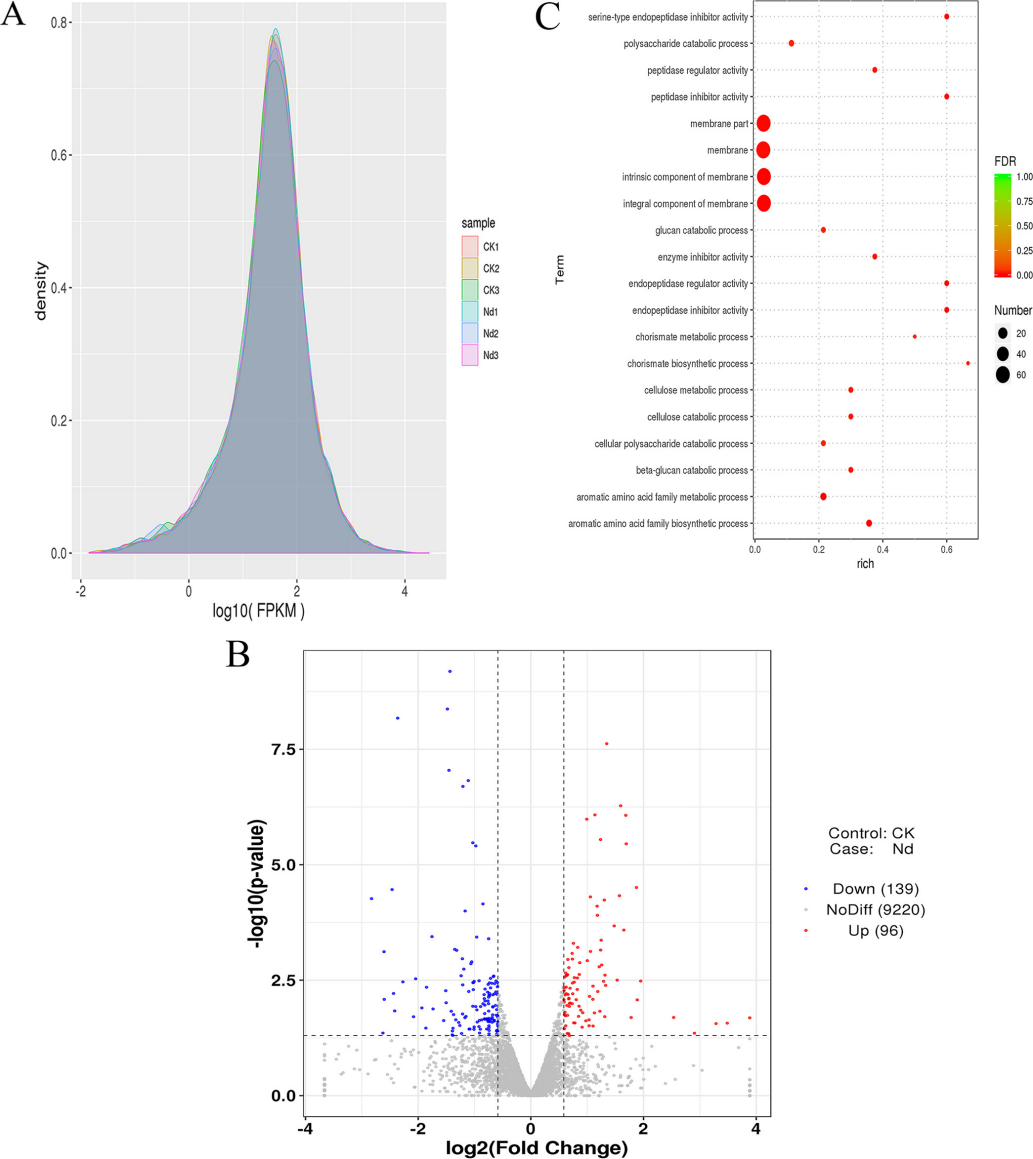
(A) FPKM density distribution. (B) Differentially expressed genes (DEGs) in L. edodes brown film cultivated on substrate containing composted sawdust (ND) and fresh sawdust (CK). Genes were considered differentially expressed when FC ≥ 1.5 or ≤ 0.667 and *P* <0.05. (C) Twenty most significant GO terms in the GO enrichment analysis of DEGs.

After Q20 screening, approximately 43,000,000 to 49,000,000 clean reads per sample passed the quality filtering, accounting for 94% of the raw reads in the cDNA libraries from the brown film of L. edodes (Table S1). The raw sequence data were submitted to NCBI Sequence Read Archive (SRA) with accession number PRJNA705065. From 70% to 79% of the quality filtered reads were mapped to L. edodes genome, approximately 84% of those were mapped to genes, and more than 96% of those to exons (Table S2). Based on the fragments per kilobase per million mapped reads (FPKM), the gene expression levels in both the CK and ND treatments were similar (Fig. 1A), implying that the data was amenable to differential expression analysis.

10.1128/mSystems.00827-21.2 TABLE S1 Mapped results of the RNA sequencing data. Download Table S1, DOCX file, 0.02 MB. Copyright © 2022 Huang et al. 2022 Huang et al. https://creativecommons.org/licenses/by/4.0/ This content is distributed under the terms of the Creative Commons Attribution 4.0 International license.

10.1128/mSystems.00827-21.3 TABLE S2 Detailed analysis of RNA-Seq mapped events. Download Table S2, DOCX file, 0.01 MB. Copyright © 2022 Huang et al. 2022 Huang et al. https://creativecommons.org/licenses/by/4.0/ This content is distributed under the terms of the Creative Commons Attribution 4.0 International license.

Among the 9,455 genes that were recovered and annotated, genes encoding a hypothetical protein, MFS general substrate transporter, cytochrome P450, alpha beta-hydrolase, P-loop containing nucleoside triphosphate hydrolase protein, kinase-like protein, and WD40 repeat-like protein were the most highly expressed (Table S3). Among the 235 differentially expressed genes (DEGs), 96 were upregulated and 139 downregulated in ND compared with CK (Fig. 1B). The upregulated DEGs included genes encoding MFS general substrate transporter, cytochrome P450, and serine protease inhibitor. The downregulated DEGs included genes encoding for a late embryogenesis abundant (LEA) domain containing protein, aldo-keto reductase, and alpha/beta hydrolase.

10.1128/mSystems.00827-21.4 TABLE S3 The expression of differential expressed genes in transcriptome. Download Table S3, XLSX file, 1.1 MB. Copyright © 2022 Huang et al. 2022 Huang et al. https://creativecommons.org/licenses/by/4.0/ This content is distributed under the terms of the Creative Commons Attribution 4.0 International license.

In the gene ontology (GO) analysis, the DEGs were assigned into 441 GO terms. In the biological processes GO category, the aromatic amino acid family metabolic and biosynthetic processes and chorismate GO terms were enriched in the ND, and the metabolic and catabolic processes of cellulose and beta-glucan and the catabolic processes of glucan, polysaccharide, and cellular carbohydrates and polysaccharides were depleted (Fig. 1C, Table S4). In the molecular functions GO category, the endopeptidase and peptidase inhibitor and regulator activities, the enzyme and serine-type endopeptidase inhibitor activities, and the phosphate acting carbon-oxygen lyase activity were enriched in ND. In the cell component category, 24 and 37 DEGs assigned into four membrane components GO terms were enriched and depleted, respectively, in the ND. In the Kyoto Encyclopedia of Genes and Genomes (KEGG) enrichment analysis, the DEGs were assigned into 28 pathways (Table S5). The phenylalanine, tyrosine and tryptophan biosynthesis (ko00400) pathway was enriched in ND.

10.1128/mSystems.00827-21.5 TABLE S4 GO analysis of DEGs in transcriptome. Download Table S4, XLSX file, 0.04 MB. Copyright © 2022 Huang et al. 2022 Huang et al. https://creativecommons.org/licenses/by/4.0/ This content is distributed under the terms of the Creative Commons Attribution 4.0 International license.

10.1128/mSystems.00827-21.6 TABLE S5 KEGG enrichment analysis of DEGs in transcriptome. Download Table S5, XLSX file, 0.01 MB. Copyright © 2022 Huang et al. 2022 Huang et al. https://creativecommons.org/licenses/by/4.0/ This content is distributed under the terms of the Creative Commons Attribution 4.0 International license.

### qPCR validation of differentially expressed genes.

The expression levels of 18 DEGs were validated using qPCR. Nine genes passed the differential abundance criteria in both RNAseq and qPCR analyses (Table 2); four out of eight genes assigned to the GO term integral component of membrane, two out of four genes assigned to the GO term aromatic amino acid family metabolic process, all the three genes assigned to the GO term catalytic activity, and none of the three genes assigned to the GO term carbohydrate metabolic process.

**TABLE 2:** The validation of differential expression in the transcriptomes of L. edodes brown film cultivated on substrate containing composted sawdust and fresh sawdust

GO term	Gene	RNAseq		qPCR	
		Fold change	*P* value	Fold change	*P* value
Integral component of membrane	**LENED_005025**	**0.46**	**0.00** ^*a*^	**0.40**	**0.03**
	**LENED_003418**	**3.02**	**0.00**	**2.84**	**<0.05**
	LENED_004710	2.26	0.00	1.99	0.11
	LENED_006239	0.35	0.01	0.69	0.10
	LENED_005737	2.34	0.02	1.26	0.02
	**LENED_010894**	**2.48**	**0.02**	**2.20**	**0.02**
	LENED_010054	0.47	0.04	0.80	0.02
	**LENED_007873**	**2.54**	**0.00**	**2.22**	**0.02**
Aromatic amino acid family metabolic process	LENED_003330	3.67	0.00	2.65	0.11
	**LENED_012514**	**2.47**	**0.00**	**1.83**	**0.02**
	LENED_009444^*b*^	2.36	0.00	0.68	0.02
	**LENED_002514**	**2.32**	**0.00**	**1.71**	**0.04**
Catalytic activity	**LENED_004417**	**1.77**	**0.00**	**1.62**	**0.02**
	**LENED_001863**	**3.23**	**0.00**	**2.23**	**0.02**
	**LENED_006399**	**0.43**	**0.00**	**0.56**	**0.02**
Carbohydrate metabolic process	LENED_007694	0.49	0.00	1.11	0.05
	LENED_009211	0.17	0.01	0.02	0.06
	LENED_001736	0.38	0.05	0.54	0.10

Genes determined as differentially expressed using both RNAseq and qRT-PCR are in bold.

a *P* value lower than 0.01.

b In the cellular component category assigned to the term integral component of membrane.

### Proteome analysis.

**FIG 2:**
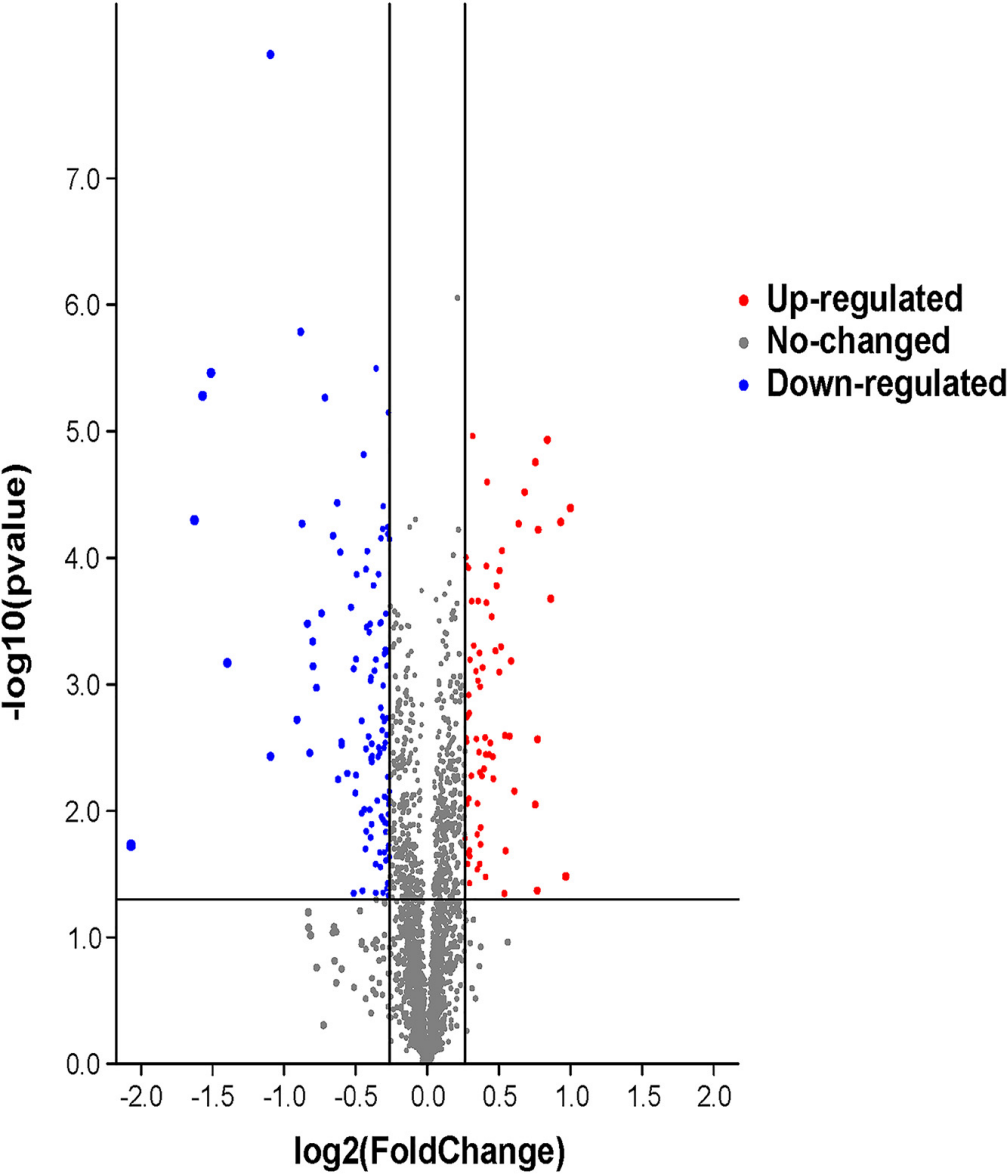
Differentially expressed proteins in L. edodes brown film cultivated on substrate containing composted sawdust (ND) and fresh sawdust (CK). Proteins were considered differentially expressed when FC ≥ 1.2 or ≤ 0.833 and *P* < 0.05.

**FIG 3:**
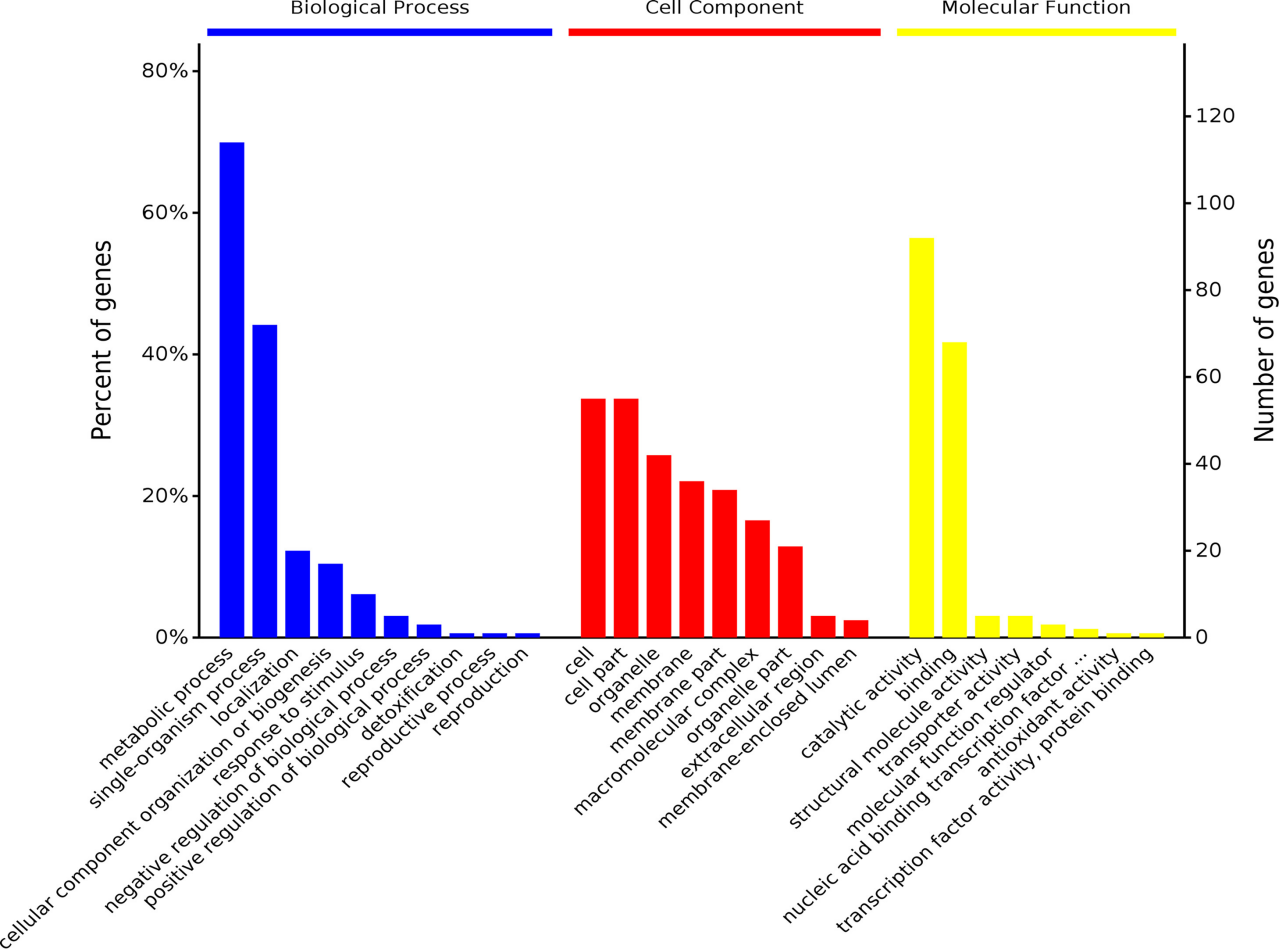
GO enrichment analysis at level 2 of differentially expressed proteins in L. edodes brown film cultivated on substrate containing composted sawdust and fresh sawdust.

**FIG 4:**
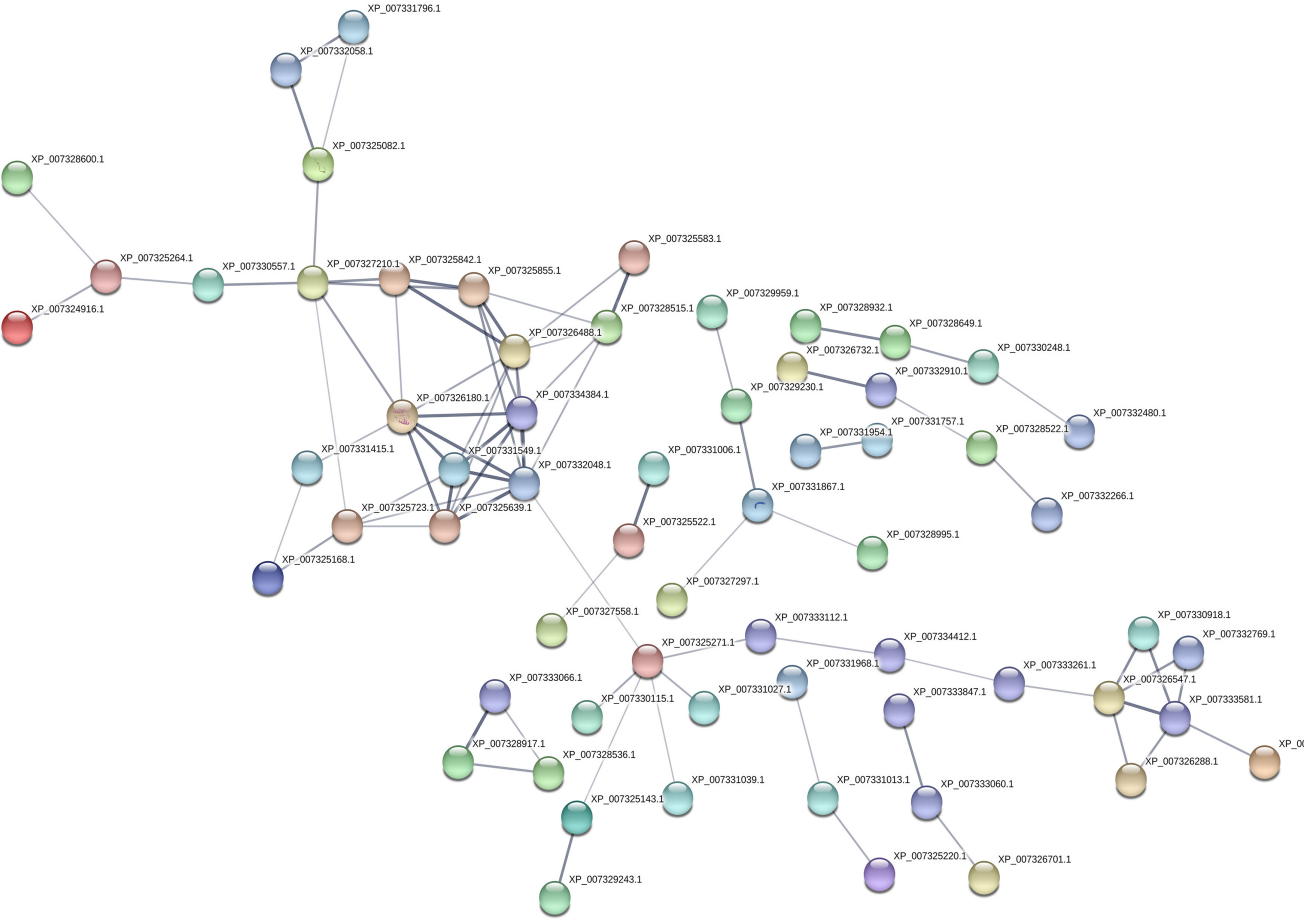
Protein-protein interaction networks of differentially expressed proteins generated with STRING (v11). Nodes represent proteins and lines represent protein-protein interactions (PPIs). Only XP-007325271.1 is an acyl carrier protein/NADP ubiquinone oxidoreductase (ACP), the others are hypothetical proteins.

In the quantitative proteomic analysis using TMT labeled peptides, 2,509 proteins and 8,407 peptides were identified in the “shiitake” database (Table S6 and S7). The 187 differentially expressed proteins included 74 upregulated proteins and 113 downregulated proteins in ND compared with CK (Fig. 2, Table S8). In the GO analysis, the highest numbers of differentially expressed proteins (DEPs) were assigned into metabolic and single-organism processes, cell and cell part, and catalytic activity and binding (Fig. 3). The GO terms hydrolase activity, acting on glycosyl bonds, and hydrolase activity, hydrolyzing O-glycosyl compounds, were depleted in the ND (*P* <0.05) (Table S9). In the KEGG enrichment analysis, the DEPs were assigned into 74 pathways in ND (Table S10). Proteins XP-007327210.1, XP-007325842.1, XP-007325855.1, XP-007328515.1, XP-007326488.1, XP-007326180.1, XP-007334384.1, XP-0073315491. XP-007332048.1, XP-007325723.1, XP-007325639.1, XP-007325271.1, XP-007326547.1, and XP-007333581.1 have the highest degree of connection, and are the key proteins in the network diagram. Among these key proteins, the others are hypothetical proteins, except that XP-007325271.1 is an acyl carrier protein/NADP ubiquinone oxidoreductase (ACP). Proteins XP-007326180.1, XP-007334384.1, XP-007331549.1, XP-007332048.1, and XP-007325639.1 all interact directly with each other (Fig. 4).

10.1128/mSystems.00827-21.7 TABLE S6 The identified proteins in the quantitative proteomic analysis. Download Table S6, XLSX file, 0.5 MB. Copyright © 2022 Huang et al. 2022 Huang et al. https://creativecommons.org/licenses/by/4.0/ This content is distributed under the terms of the Creative Commons Attribution 4.0 International license.

10.1128/mSystems.00827-21.8 TABLE S7 The identified peptides in the quantitative proteomic analysis. Download Table S7, XLSX file, 2.6 MB. Copyright © 2022 Huang et al. 2022 Huang et al. https://creativecommons.org/licenses/by/4.0/ This content is distributed under the terms of the Creative Commons Attribution 4.0 International license.

10.1128/mSystems.00827-21.9 TABLE S8 The DEPs in the proteome. Table S7 The DEPs in the proteome. Download Table S8, XLSX file, 0.1 MB. Copyright © 2022 Huang et al. 2022 Huang et al. https://creativecommons.org/licenses/by/4.0/ This content is distributed under the terms of the Creative Commons Attribution 4.0 International license.

10.1128/mSystems.00827-21.10 TABLE S9 GO analysis of DEPs in proteome. Download Table S9, XLSX file, 0.1 MB. Copyright © 2022 Huang et al. 2022 Huang et al. https://creativecommons.org/licenses/by/4.0/ This content is distributed under the terms of the Creative Commons Attribution 4.0 International license.

10.1128/mSystems.00827-21.11 TABLE S10 KEGG enrichment analysis of DEPs in proteome. Download Table S10, XLSX file, 0.02 MB. Copyright © 2022 Huang et al. 2022 Huang et al. https://creativecommons.org/licenses/by/4.0/ This content is distributed under the terms of the Creative Commons Attribution 4.0 International license.

### CAZymes analysis.

In the transcriptome, genes annotated as carbohydrate-active enzymes (CAZymes) accounted for 7.2% of the differentially expressed genes. One carbohydrate esterase (CE), one glycosyltransferase (GT), and four auxiliary activities (AA) genes were upregulated, and five glycoside hydrolase (GH), three CE, one carbohydrate-binding module (CBM), one GT, and one AA genes were downregulated (Table 3). In the proteome, proteins annotated as CAZymes accounted for 15.5% of the differentially expressed proteins. Three GH proteins and one AA protein were upregulated, and 14 GH, four CE, six AA ,and one polysaccharide lyase (PL) proteins were downregulated (Table 3).

**TABLE 3:** Differentially expressed genes and proteins annotated to CAZymes in the transcriptomes of L. edodes brown film cultivated on substrate containing composted sawdust (ND) and fresh sawdust (CK)

Gene ID/ Protein accession	CAZymes category	Fold change	*P* value	Gene ID/ Protein accession	CAZymes category	Fold change	*P* value
*LENED_012311*	AA	3.7	0.01	*LENED_007694*	GH	0.49	0.00*
*LENED_010054*	AA	0.47	0.04	*LENED_003770*	GH	0.59	0.02
*LENED_009769*	AA	1.61	0.05	*LENED_001736*	GH	0.38	0.05
*LENED_005504*	AA	2.79	0.00*	A0A1Q3ET82	GH	0.56	0.00*
*LENED_001847*	AA	1.82	0.00*	A0A1Q3ESS7	GH	0.78	0.00*
A0A1Q3EPZ8	AA	0.74	0.01	A0A1Q3EQ81	GH	0.38	0.00*
A0A1Q3ENQ4	AA	1.7	0.04	A0A1Q3EQ40	GH	0.57	0.00*
A0A1Q3ENB3	AA	0.81	0.00*	A0A1Q3ELT5	GH	0.66	0.00*
A0A1Q3EJ91	AA	0.57	0.00*	A0A1Q3EJ18	GH	0.76	0.00*
A0A1Q3EJ75	AA	0.73	0.01	A0A1Q3EGJ4	GH	0.61	0.00*
A0A1Q3EB49	AA	0.83	0.01	A0A1Q3EGI8	GH	0.35	0.00*
A0A1Q3E0C3	AA	0.54	0.00*	A0A1Q3E897	GH	0.79	0.00*
*LENED_004424*	CBM	0.45	0.02	A0A1Q3E7U3	GH	0.78	0.00*
*LENED_009143*	CE	0.41	0.03	A0A1Q3E2W0	GH	1.2	0.02
*LENED_008379*	CE	0.6	0.04	A0A1Q3E1P4	GH	1.52	0.01
*LENED_004554*	CE	1.99	0.00*	A0A1Q3E184	GH	1.32	0.00*
*LENED_001825*	CE	0.42	0.00*	A0A1Q3E0I6	GH	0.75	0.00*
A0A1Q3EMX7	CE	0.82	0.01	A0A1Q3DZD5	GH	0.78	0.04
A0A1Q3EKQ2	CE	0.74	0.00*	A0A1Q3DYA6	GH	0.65	0.01
A0A1Q3EEB1	CE	0.71	0.00*	A0A1Q3DUH1	GH	0.79	0.00*
A0A1Q3E245	CE	0.63	0.00*	*LENED_010922*	GT	0.61	0.00*
*LENED_010692*	GH	0.62	0.02	*LENED_009815*	GT	1.67	0.01
*LENED_009211*	GH	0.16	0.01	A0A1Q3EEP3	PL	0.47	0.00*

*, *P* value lower than 0.01.

### Correlation between transcriptome and proteome.

**FIG 5:**
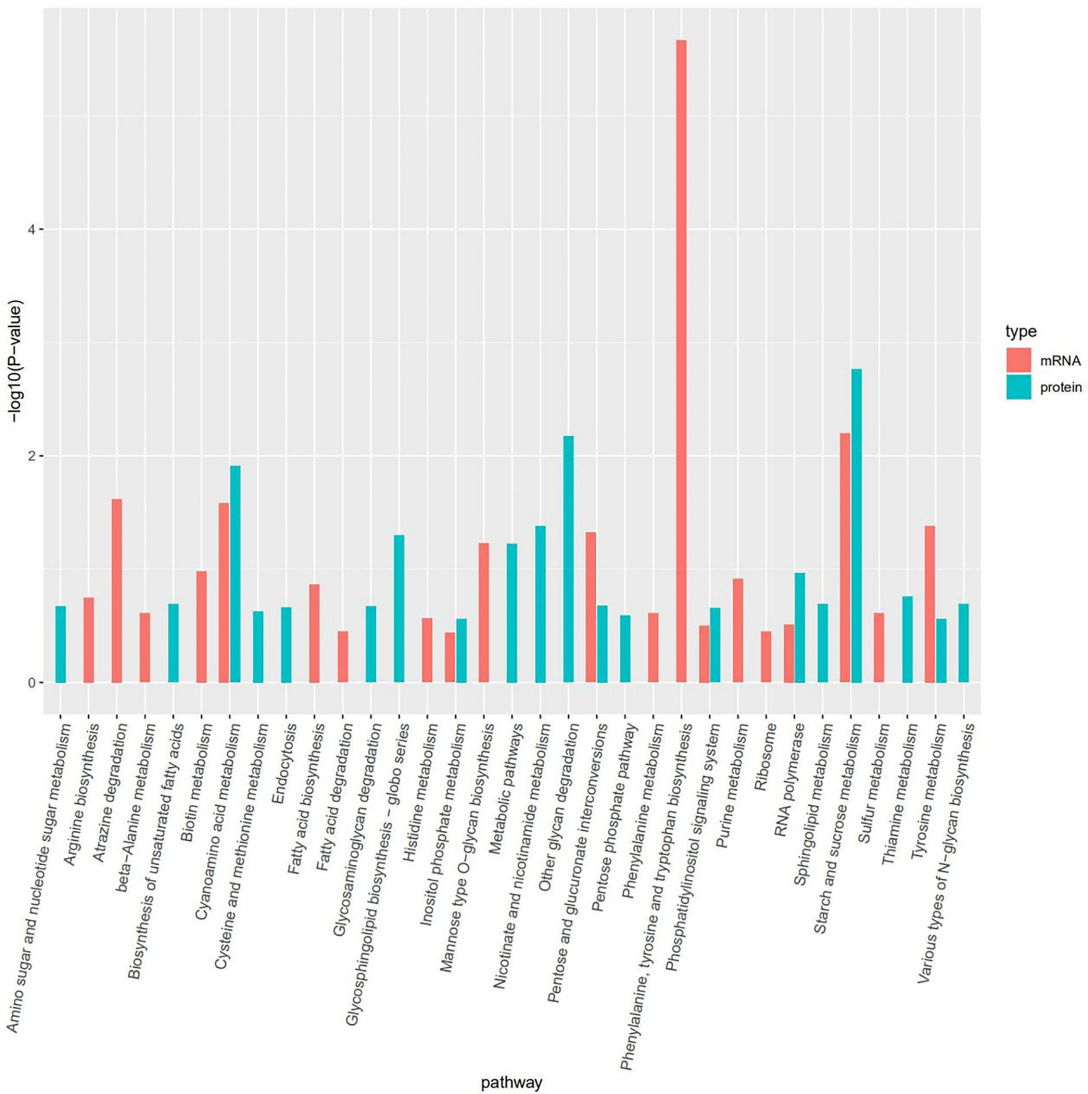
KEGG pathway analyses of differentially expressed genes (mRNA) and proteins in L. edodes brown film cultivated on substrate containing composted sawdust and fresh sawdust.

The Pearson correlation coefficient between transcriptome and proteome was 0.072. When the results of proteome and transcriptome enrichment were collated, it was found that there were seven pathways in common among the TOP 20 KEGG pathway in the two omics, respectively, that are cyanoamino acid metabolism, inositol phosphate metabolism, pentose and glucuronate interconversions, phosphatidylinositol signaling system, RNA polymerase, starch and sucrose metabolism, and tyrosine metabolism pathways (Fig. 5). Three pairs of differentially expressed proteins and genes were detected in both the proteome and transcriptome; LENED_010224 and the corresponding protein A0A1Q3ELU5 and LENED_004417 (A0A1Q3E6U4) were upregulated in both, and LENED_009984 (A0A1Q3ELG3) was upregulated in the proteome and downregulated in the transcriptome. In the GO analysis, A0A1Q3ELU5 and A0A1Q3E6U4 were assigned into endopeptidase inhibitor activity and oxidoreductase activity (Table S7). Three pathways with high significance (*P*-value <0.05), namely, tyrosine metabolism, fatty acid degradation and glycolysis/gluconeogenesis, were obtained by KEGG enrichment analysis. And A0A1Q3E6U4 is involved in the enrichment of these three pathways.

### Enzyme assay.

**FIG 6:**
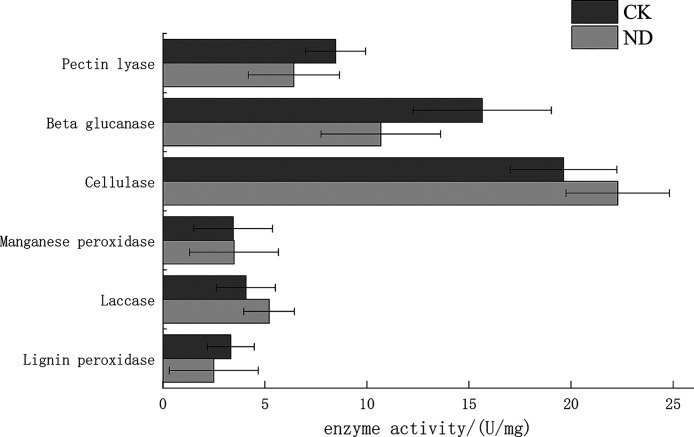
Pectin lyase, beta glucanase, cellulase, manganese peroxidase, laccase, and lignin peroxidase activities in L. edodes brown film cultivated on substrate containing composted sawdust (ND) and fresh sawdust (CK).

In assaying the effect of sawdust composting on the lignocellulose degradation related enzymatic activity on brown film of L. edodes, the activity of pectin lyase ranged from approximately 5 to 10 U mL^−1^, the activity of beta glucanase from below 10 to approximately 20 U mL^−1^, the activity of cellulase from approximately 16 to 25 U mL^−1^, and the activities of manganese peroxidase, laccase, and lignin peroxidase were below approximately 7 U mL^−1^ (Fig. 6). The differences in enzyme activities in CK and ND were not statistically significant.

## DISCUSSION

Brown film formation is an important step during the growth of L. edodes and associated with the quality of the fruiting body. We studied L. edodes brown film formation using combined transcriptome and proteome analyses. The growth substrate, most commonly sawdust based, influences the brown film formation and thus directly affects the quality of the fruiting body. Compared with the traditional cultivation on natural logs, growing L. edodes on sawdust based synthetic logs results in poorer quality of the fruiting bodies (15). In the composting of lignocellulose-rich material, e.g., sawdust, microbial degradation of polymers releases soluble organic intermediates (16). In agreement, our study showed the lignin, hemicellulose, and cellulose contents were lower in the composted (ND) than in the not composted (CK) sawdust. However, the minor differences in the activities of lignocellulose degrading enzymes were not statistically significant. The composting accompanied changes may affect the growth of fungi; the yield of Pleurotus ostreatus was higher on cultivation substrate with composted poplar sawdust than with not composted sawdust (17, 18). Similarly, in our study the time of brown film formation was shorter, and the mycelium growth rate was higher on the growth substrate that included composted sawdust. The faster growth rate in ND than in CK may have been due to the higher nitrogen content that has been shown to result in higher biomass production and growth efficiency of saprotrophic fungi (18, 19). In addition, the brown film formation is the key period for the synthesis and accumulation of polysaccharides in L. edodes (4), possibly explaining the higher polysaccharide content in L. edodes grown on composted sawdust substrate.

Growing L. edodes on composted sawdust containing substrate affected the expression of genes. We detected 235 and 187 DEGs and DEPs, respectively, in ND compared with CK. Out of the DEGs and DEPs, 7.2% and 15.5%, respectively, were annotated as carbohydrate-active enzymes (CAZymes) that participate in the synthesis of polysaccharides and the hydrolysis of cell wall and play an important role in substrate degradation (20). Approximately half of the differentially expressed CAZymes were annotated as GHs that hydrolyze glycosidic bonds in carbohydrates or between carbohydrates and non-carbohydrates (21). Most of the differentially expressed GHs and other CAZymes were downregulated in L. edodes growing on composted sawdust containing substrate, possibly due to the lower hemicellulose and cellulose contents in the composted sawdust.

Lignin peroxidase (Lip) has strong oxidizing power, and the main oxidizing substrate is aromatic compounds (22) and laccase has a low substrate specificity and can oxidize a range of compounds (23). After fermentation, lignin produced free aromatic compounds and a variety of different kinds of small molecules. This may be the reason for the higher activity of laccase and lower activity of LiP in the ND group in this research. The high activities of cellulase may be the addition of wheat bran in substrate, and the main component of wheat bran is cellulose. Therefore, to degrade cellulose, the activity of cellulase must be improved. While the activity of cellulase in ND was higher, perhaps the reason is that the brown film formation time of CK is not as long as ND, so they are not at the same stage. However, there were no statistically significant differences in enzyme activities.

The growth substrate may affect the exchange of nutrients and the degradation of harmful substances. The major facilitator superfamily (MFS) transporters include nearly one fourth of the transporters encoded in microbial genomes (24). MFS transporters can secrete fungal antibiotics, toxins, and other metabolites (25, 26), playing an important role in transporting monosaccharides, polysaccharides, amino acids, peptides, vitamins, and many other small molecules (27, 28). In our study, the genes encoding MFS general substrate transporter and cytochrome P450 were among the highly expressed genes and upregulated in ND compared with CK, suggesting that more material was transported at this stage. Fungal cytochrome P450 enzyme systems mediate bioconversions of polycyclic aromatic hydrocarbons, sterols, and alkanes (29–31), and the accumulation of these substances promotes the expression of cytochrome P450 (30, 32–34). Cytochrome P450 was suggested to be involved in the stipe elongation of L. edodes (35, 36). In agreement with the higher growth rate, genes encoding cytochrome P450 were upregulated in ND compared with CK.

In gene expression, mRNA abundance is governed by transcription and mRNA degradation, and protein abundance by translation, the main controller of protein abundance, and protein degradation (37, 38), which may explain the poor correlation between transcriptome and proteome. However, seven KEGG pathways were differentially expressed based on both transcriptome and proteome analyses. The *P*-value of the starch and sucrose metabolism was lowest in both two omics. We think changes in this metabolism were likely connected to the differences in cellulose and hemisellulose contents of the substrates and linked to the higher polysaccharide contents of L. edodes grown on composted substrate. The higher growth rate on composted substrate was possibly reflected in the differential expression of RNA polymerase. In addition, the inositol phosphate metabolism and the connected phosphatidylinositol signaling system and pentose and glucuronate interconversions are involved in growth and metabolic adaptation (39), and their differential expression was also possibly related to the difference in growth rate. Similarly, the differential expression of cyanoamino acid metabolism and its component tyrosine metabolism pathway may have been linked to the difference in nitrogen content between composted and not composted substrate.

### Summary.

We applied transcriptomic and proteomic approaches to study L. edodes cultivated on substrates with fresh (CK) and composted (ND) sawdust, focusing on the brown film formation stage. The time of brown film formation was shorter and the mycelium growth rate was higher on the growth substrate that included composted sawdust, suggesting that using composted substrate may decrease the cultivation time of L. edodes. In addition, the crude polysaccharide content in the brown film was higher, implying higher quality. The faster growth rate in ND than in CK may have been due to the higher nitrogen content in ND. Most of the differentially expressed genes annotated to carbohydrate active enzymes were downregulated in L. edodes growing on composted sawdust containing substrate, possibly due to the lower hemicellulose and cellulose contents in the composted sawdust. The genes encoding MFS general substrate transporter and cytochrome P450 were upregulated in ND compared with CK, suggesting that more material was transported at this stage. Changes in the KEGG pathways were likely connected to the differences in cellulose, hemisellulose, and nitrogen contents of the substrates, and to the higher growth rate and polysaccharide contents of *L. edodes* grown on composted substrate.

## MATERIALS AND METHODS

### Substrate preparation and analysis.

Fresh Fagus sylvatica sawdust with water content adjusted to 65% was composted in 2.0 m × 1.8 m × 1.5 m piles in an open space for 90 days. The piles were turned over during the heating and high temperature periods of the composting process. Samples were taken on day 0 and day 90 from the upper, middle, and lower sections of the sawdust pile. The 500 g samples were stored at –20°C. The sawdust samples were homogenized and passed through a 40-mesh sieve prior the analyses. Then, 10 g of sawdust was suspended in 90 mL of sterilized distilled water and left to stand 2 h at 25°C, after which pH was measured using a glass electrode pH meter (FiveGo, Mettler Toledo, Greifensee, Switzerland). The contents of cellulose, hemicellulose, and lignin were determined using the Van Soest method (40, 41). Total nitrogen and organic carbon contents were determined using the Kjeldahl method and the potassium dichromate volumetric method, respectively.

### *L. edodes* cultivation.

The rest of the sawdust samples was used for L. edodes cultivation. A mixture of 80% sawdust, 18% wheat bran, 1% sucrose, and 1% calcium carbonate was suspended in water in a 1:1.5 ratio (w:vol). The substrates containing either fresh (CK) or composted (ND) sawdust were packed into mushroom culture packages each weighing approximately 1 kg, sterilized, and cooled to room temperature. Each treatment included 40 culture packages. L. edodes ACCC50302 was obtained from the Agricultural Culture Collection of China (ACCC). Precultured L. edodes was inoculated onto two sides of the culture packages. To obtain a uniform spread of the hypha in the substrate, packages were kept at 20°C to 24°C and 65 % to 70% relative humidity in the dark. After the mycelium had colonized the medium completely, the culture packages were kept at 12°C to 22°C and 85% to 90% relative humidity in dark. At the brown film formation stage, the mycelia from three randomly selected replicate samples for the extraction of RNA, protein, and polysaccharide were collected, mixed thoroughly, frozen immediately in liquid nitrogen, and stored at −80°C.

### RNA extraction, cDNA library construction, and sequencing.

RNA was extracted using the TRIzol Reagent (Invitrogen Life Technologies, USA). The concentration and quality of extracted RNA were determined using a NanoDrop spectrophotometer (Thermo Scientific). The integrity of the RNA was assessed with 1% agarose by electrophoresis.

Sequencing libraries were generated using the TruSeq RNA Sample Preparation Kit (Illumina, San Diego, CA, USA) (42). Briefly, mRNA was purified from three μg RNA using poly-T oligo-attached magnetic beads (43). Fragmentation was carried out using divalent cations under elevated temperature in an Illumina proprietary fragmentation buffer. First strand cDNA was synthesized using random oligonucleotides and SuperScript II. Second strand cDNA was synthesized using DNA polymerase I and RNase H. Remaining overhangs were converted into blunt ends via exonuclease/polymerase activities and the enzymes were removed. After acetylation of the 3′ ends of the DNA fragments, Illumina PE adapter oligonucleotides were ligated to prepare for hybridization (44). To select cDNA fragments of the preferred 200 bp in length, the library fragments were purified using the AMPure XP system (Beckman Coulter, Beverly, CA, USA). DNA fragments with ligated adaptors on both ends were selectively enriched using Illumina PCR Primer Cocktail in a 15 cycle PCR. Products were purified using AMPure XP system and quantified using the Agilent high sensitivity DNA assay on a Bioanalyzer 2100 system (Agilent). The sequencing library was sequenced on a Hiseq platform (Illumina) at Shanghai Personal Biotechnology Co. Ltd, China, and three repeats were done of each treatment.

### Expression analysis.

High-quality clean reads were aligned to the assembled transcriptome using Bowtie2 (http://bowtie-bio.sourceforge.net/bowtie2/manual.shtml), and compared with the L. edodes reference genome (*Lentinula_edodes.*Lened_assembly 01.dna.toplevel.fa) using Tophat2. The Q20 and Q30 values, GC content, and sequence duplication level of clean reads were calculated. The alignment counts were obtained using RSEM package (http://deweylab.github.io/RSEM/). The read count value of each gene was compared by HTSeq statistics as the original expression amount of the gene. Reads count was positively correlated with the true gene expression level, gene length, and sequencing depth. In order to make the gene expression levels of different genes and different samples comparable, FPKM was used to normalize the expression levels. FPKM is used to calculate the number of fragments from a gene per million fragments that two reads can compare to the same transcript. In the differential expression analysis using the EBSeq R package (45, 46), genes were considered differentially expressed when fold change (FC) ≥ 1.5 or ≤ 0.667 and *P* < 0.05. *P* values were adjusted for multiple testing using the procedure described by Yang (47).

### Functional and pathway analysis of DEGs.

Transcripts were functionally annotated against Universal Protein Knowledgebase (UniProt) (48), gene ontology (GO) (49), eggNOG (50), and KEGG databases (51) using BLAST (52). GO analysis was performed on the annotated genes, mainly including cellular component, molecular function, and biological processes. KEGG pathway enrichment analysis of differentially expressed genes was performed with KEGG biological pathways database (http://www.genome.jp) to understand the function of differentially expressed genes. KEGG orthology (KO) analysis of unigenes was performed using the KOBAS 2.0 web server (http://kobas.cbi.pku.edu.cn/) (53).

### Protein extraction and peptide labeling.

Samples were ground into powder in liquid nitrogen with a mortar and pestle. Proteins were extracted using SDT (4% [wt/vol] SDS, 100 mM Tris/HCl pH 7.6, 0.1m DTT) pyrolysis (54), and quantified using a BCA assay. Appropriate amount of protein for trypsin digestion was extracted using filter-aided sample preparation (FASP) (54). The enzymatic hydrolysis peptide was desalted by C18 Cartridge. The peptide was freeze-dried and soluble by adding 40 μL dissolution buffer. The peptide was quantified by OD280.

### LC-MS/MS analysis.

LC-MS/MS was done using a Thermo Scientific Easy nLC HPLC connected to a Q-Exactive mass spectrometer. The labeled peptides were loaded into a Thermo Scientific EASY C18 sample loading column (100 μm*2 cm, 5 μm) and then separated using a Thermo scientific EASY C18 analytical column (75 μm*10cm, 3 μm) at a flow rate of 250 nl min^−1^ for 60 min. The mass spectrometer was operated in positive mode. In the MS scan, precursor ion scanning range was 300 to 1,800 *m/z*, resolution of primary mass spectrum was 70,000 at *m/z* 200, automatic gain control (AGC) target was 3e6, maximum injection time was 10 ms, and the dynamic exclusion time was 40.0 s. Ten fragment maps (MS2 scan) were collected after each full MS scan. The MS/MS parameters included HCD activation type, isolation window of 2 *m/z*, resolution of 17,500 at 200 *m/z*, normalized collision energy of 30 eV, and underfill ratio of 0.1%. The Mascot2.2 and Proteome Discoverer1.4 were used for “uniprot_shiitake” database identification and quantitative analysis. Each treatment was repeated three times for proteome sequencing. Proteins were considered differentially expressed when fold change (FC) ≥ 1.2 or ≤ 0.833 and *P* <0.05. GO analysis and KEGG pathway analysis of differentially expressed proteins (DEPs) were done using Omicsbean (55). Protein-protein interaction network analysis was conducted based on the information in the STRING database v11 (http://string-db.org/) (56).

### CAZymes analysis.

The genes and proteins related to carbohydrate-active enzymes (CAZymes) that were differently expressed in the transcriptome and proteome of L. edodes were analyzed using the CAZymes Analysis Toolkit (CAT) and the carbohydrate enzyme database (http://www.cazy.org) (57, 58).

### Correlation analysis between transcriptome and proteome.

The differential expression and enrichment results of proteins and genes were collated, the correlation differentially expressed genes and proteins were screened out, and then the associated differentially expressed proteins were analyzed by GO and KEGG analysis.

### qRT-PCR validation of RNA-Seq data.

Eighteen differentially expressed genes were selected for validation by quantitative real-time PCR (qRT-PCR). qRT-PCR was performed on a CFX96 Real-Time System (BIO-RAD) with SYBR green as the fluorescent dye according to the manufacturer’s protocol (59). qRT-PCR validation included three biological replicates with three technical replicates and GAPDH as the internal control gene as previously described (60). Genes were considered differentially expressed when FC ≥ 1.5 or ≤ 0.667 and *P* <0.05.

### Enzyme assay.

To extract extracellular enzymes from the brown film, 10 g wet weight growth medium was suspended in 200 mL of 50 mM sodium acetate buffer (pH 4.8) and centrifuged at 180 rpm for 1 h. The activities of Lip, laccase, manganese peroxidase (MnP), and cellulase were determined as previously described (61, 62). To determine the activity of pectin lyase, 1.8 mL pectin substrate solution and 0.2 mL crude enzyme solution were mixed and incubated in a water bath at 50°C for 30 min. Then, 3 mL DNS reagent was added and the mixture was incubated in a boiling water bath for 10 min. Prior measuring the absorbance value at 560 nm, the mixture was diluted 5-fold with ddH_2_O. The sample enzyme activity was interpolated from a standard curve. The β-glucanase activity was determined as described in Table 4. The enzyme activity was defined as units per mL (U mL^−1^) where U is the amount of enzyme that catalyzes the conversion of 1 μmol of substrate.

**TABLE 4:** Steps for the determination of β-glucanase activity

Sample	Control
Substrate 1.8 mL	Substrate 1.8 mL
Preheat at 50°C for 3 min	Preheat at 50°C for 3 min
Enzyme solution 0.2 mL	Hydrolysis at 50°C for 10 min
Mix	DNS solution 3 mL
Hydrolysis at 50°C for 10 min	Mix
DNS solution 3 mL	Enzyme solution 0.2 mL
Mix	Mix
Boil in a boiling water bath for 10 min	Boil in a boiling water bath for 10 min
Cool and allow to 15 mL	Cool and allow to 15 mL
