# Supplementary material for: Cultivating Lentinula edodes on Substrate Containing Composted Sawdust Affects the Expression of Carbohydrate and Aromatic Amino Acid Metabolism-Related Genes
Source: mSystems. 2022 Feb 22;7(1):e00827-21. doi: 10.1128/msystems.00827-21 (PMC8862593; doi:10.1128/msystems.00827-21)
Supplement: TABLE S1 [file msystems.00827-21-st001.docx]

Table S1. Mapped results of the RNA sequencing data.

| **Sample** | **Useful Reads** | **Map Events Count** | **Total Mapped** | | **Multiple Mapped** | | **Uniquely Mapped** | |
| --- | --- | --- | --- | --- | --- | --- | --- | --- |
|  |  |  | **Reads** | **%** | **Reads** | **%** | **Reads** | **%** |
| CK1 | 48,773,958 | 36,467,649 | 36,957,402 | 75.77 | 489,753 | 1.33 | 36,467,649 | 98.67 |
| CK2 | 45,596,710 | 35,212,003 | 35,702,337 | 78.30 | 490,334 | 1.37 | 35,212,003 | 98.63 |
| CK3 | 43,362,614 | 30,178,342 | 30,573,560 | 70.51 | 395,218 | 1.29 | 30,178,342 | 98.71 |
| ND1 | 43,558,996 | 33,876,138 | 34,330,919 | 78.81 | 454,781 | 1.32 | 33,876,138 | 98.68 |
| ND2 | 48,854,422 | 37,115,224 | 37,624,090 | 77.01 | 508,866 | 1.35 | 37,115,224 | 98.65 |
| ND3 | 43,548,238 | 32,810,574 | 33,255,855 | 76.37 | 445,281 | 1.34 | 32,810,574 | 98.66 |
