# Supplementary material for: Cultivating Lentinula edodes on Substrate Containing Composted Sawdust Affects the Expression of Carbohydrate and Aromatic Amino Acid Metabolism-Related Genes
Source: mSystems. 2022 Feb 22;7(1):e00827-21. doi: 10.1128/msystems.00827-21 (PMC8862593; doi:10.1128/msystems.00827-21)
Supplement: TABLE S2 [file msystems.00827-21-st002.docx]

Table S2. Detailed analysis of RNA-Seq mapped events.

| **Sample** | **Map Events** | **Mapped to Gene** | | **Mapped to InterGene** | | **Mapped to Exon** | |
| --- | --- | --- | --- | --- | --- | --- | --- |
|  |  | **count** | **%** | **count** | **%** | **count** | **%** |
| CK1 | 36,467,649 | 30,814,838 | 84.50% | 5,652,811 | 15.50% | 29,804,286 | 96.72% |
| CK2 | 35,212,003 | 29,656,922 | 84.22% | 5,555,081 | 15.78% | 28,677,650 | 96.70% |
| CK3 | 30,178,342 | 25,342,341 | 83.98% | 4,836,001 | 16.02% | 24,521,856 | 96.76% |
| ND1 | 33,876,138 | 28,539,966 | 84.25% | 5,336,172 | 15.75% | 27,599,789 | 96.71% |
| ND2 | 37,115,224 | 31,273,087 | 84.26% | 5,842,137 | 15.74% | 30,228,738 | 96.66% |
| ND3 | 32,810,574 | 27,567,860 | 84.02% | 5,242,714 | 15.98% | 26,679,926 | 96.78% |
